# Identification of Novel Chromosomal Aberrations Induced by ^60^Co-γ-Irradiation in Wheat-*Dasypyrum villosum* Lines

**DOI:** 10.3390/ijms161226134

**Published:** 2015-12-14

**Authors:** Jie Zhang, Yun Jiang, Yuanlin Guo, Guangrong Li, Zujun Yang, Delin Xu, Pu Xuan

**Affiliations:** 1 Institute of Biotechnology and Nuclear Technology Research, Sichuan Academy of Agricultural Sciences, Chengdu 610061, Sichuan, China; jade_chang@aliyun.com (J.Z.); jiangyun20000@sohu.com (Y.J.); gylin63@aliyun.com (Y.G.); 2 School of Life Science and Technology, University of Electronic Science and Technology of China, Chengdu 610054, China; ligr28@uestc.edu.cn (G.L.); yangzujun@uestc.edu.cn (Z.Y.); 3 Department of Cell Biology and Genetics, Zunyi Medical University, Zunyi 563000, Guizhou, China; xudelin@zmc.edu.cn; 4 Institute of Agro-Products Processing Science and Technology, Sichuan Academy of Agricultural Sciences, Chengdu 610066, Sichuan, China

**Keywords:** irradiation, wheat, *Dasypyrum villosum*, chromosomal aberrations, ND-FISH

## Abstract

Mutations induced by radiation are widely used for developing new varieties of plants. To better understand the frequency and pattern of irradiation-induced chromosomal rearrangements, we irradiated the dry seeds of Chinese Spring (CS)-*Dasypyrum villosum* nullisomic-tetrasomic (6A/6D) addition (6V) line (2*n* = 44), WD14, with ^60^Co-γ-rays at dosages of 100, 200, and 300 Gy. The M_0_ and M_1_ generations were analyzed using Feulgen staining and non-denaturing fluorescence *in situ* hybridization (ND-FISH) by using oligonucleotide probes. Abnormal mitotic behavior and chromosomes with structural changes were observed in the M_0_ plants. In all, 39 M_1_ plants had structurally changed chromosomes, with the B genome showing the highest frequency of aberrations and tendency to recombine with chromosomes of the D genome. In addition, 19 M_1_ plants showed a variation in chromosome number. The frequency of chromosome loss was considerably higher for 6D than for the alien chromosome 6V, indicating that 6D is less stable after irradiation. Our findings suggested that the newly obtained γ-induced genetic materials might be beneficial for future wheat breeding programs and functional gene analyses.

## 1. Introduction

Bread wheat (*Triticum aestivum* L.) is one of the most important agricultural products, accounting for 20% of global human caloric intake [1]. However, artificial selection and domestication of wheat breeding programs tend to decrease the biological diversity, thereby decreasing the resistance of varieties to various biotic and abiotic stresses and leading to heavy losses in yield. Therefore, broadening the biological diversity of current wheat varieties is necessary to increase their resistance to agricultural stresses.

*Dasypyrum villosum* (L.) (2*n* = 14, VV), a species related to wheat, is resistant to biotic causal agents of wheat diseases and abiotic stress [2]. The durable powdery mildew resistance gene, *Pm21*, was shown to be located on the short arm of chromosome 6V (6VS) of *D. villosum* [3,4]. No obvious negative agronomic traits are known to be associated with this chromosome. In wheat breeding, 6VS.6AL and 1RS.1BL are considered the most widely used translocations.

Gamma irradiation is an effective way to induce mutations and to broaden crop genetic variability. Since 1956, Sears transferred gene from *Aegilops umbellulata* (Zhuk.) to wheat genetic background [5], genetic transfer has been widely used in wheat breeding programs and for the creation of translocations to develop novel wheat genetic resources. To date, some wheat cultivars [6,7,8] have been successfully bred by incorporating ^60^Co-γ-induced mutations. Many beneficial traits have been transferred from the genomes of alien species to those of wheat by ^60^Co-γ [5,9,10,11,12,13]. Moreover, translocations and deletions induced by gamma rays have been useful in mapping and cloning target genes [13,14]. Most previous studies [5,9,10,11,12,13] mainly focused on alien chromosomes that were studied using cytological procedures such as C–banding and genomic *in situ* hybridization methods. However, cytogenetic detection is generally time-consuming and labor-intensive. Given that non-denaturing florescence *in situ* hybridization (ND-FISH) involves the use of oligonucleotides as probes, it is a novel and efficient technique to identify chromosomes [15].

In the present study, we provide a new insight into the breakage and refusion of wheat and *D. villosum* chromosomes. We showed that gamma rays produced a higher frequency of breakage in B genome chromosomes that also tended to recombine with the D genome, and that chromosome 6D was less stable after irradiation than the alien chromosome 6V. Further, we obtained 58 novel ^60^Co-γ-induced chromosomally altered plants by using ND-FISH. These new chromosomal structural variant lines and deletion lines could be used as new genetic resources for wheat breeding.

## 2. Results

### 2.1. FISH Pattern of the WD14 Chromosomes

**Figure 1:**
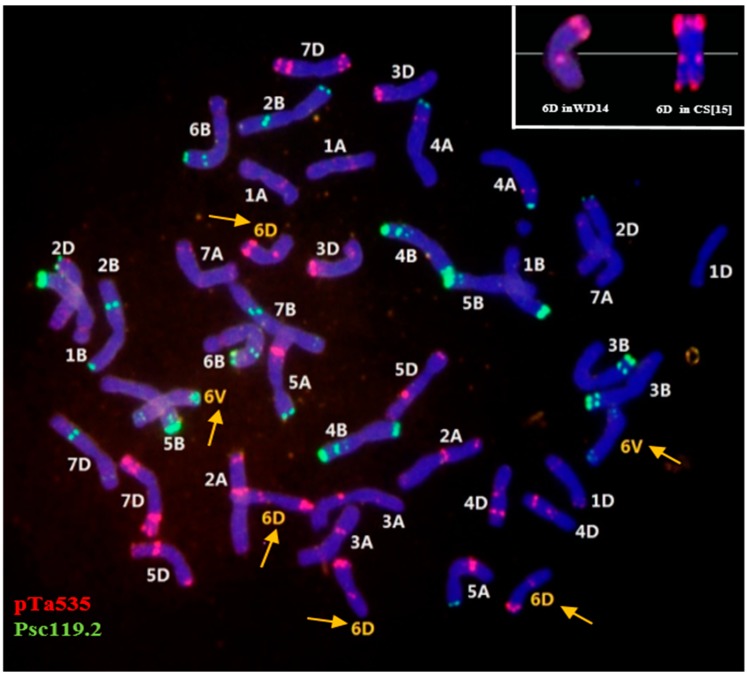
Fluorescence *in situ* hybridization was performed on mitotic chromosomes of WD14 by using oligo-pTa535-1 (red) and oligo-pSc119.2-1 (green) as probes. Yellow arrows indicate the four 6D chromosomes and two alien chromosomes 6V from *Dasypyrum villosum*. Chromosomes were counterstained with 4′-6-diamidino-2-phenylindole (blue).

The CS-*D. villosum* nullisomic-tetrasomic (6A/6D) addition (6V) line (2*n* = 44) that contained four 6D chromosomes and two 6V chromosomes without 6A was selected from the CS-*D*. *villosum* addition (6V) line#3 (2*n* = 44) and designated asWD14. The chromosomes of WD14 were analyzed using multi-color FISH by using oligo-pTa535-1 and oligo-pSc119.2-1 as probes. These probes could distinguish the chromosomes of A, B, and D genomes. The signal patterns on each chromosome were consistent with those reported by Tang *et al.* [15] with the exception of the patterns on 6D. The telomeric end of 6DS in WD14 did not show any signal of pTa535-1, whereas the end of 6DS in CS showed a significant signal [15] (Figure 1).

### 2.2. Investigation of Mitotic Metaphase Chromosomes in M_0_

**Figure 2:**
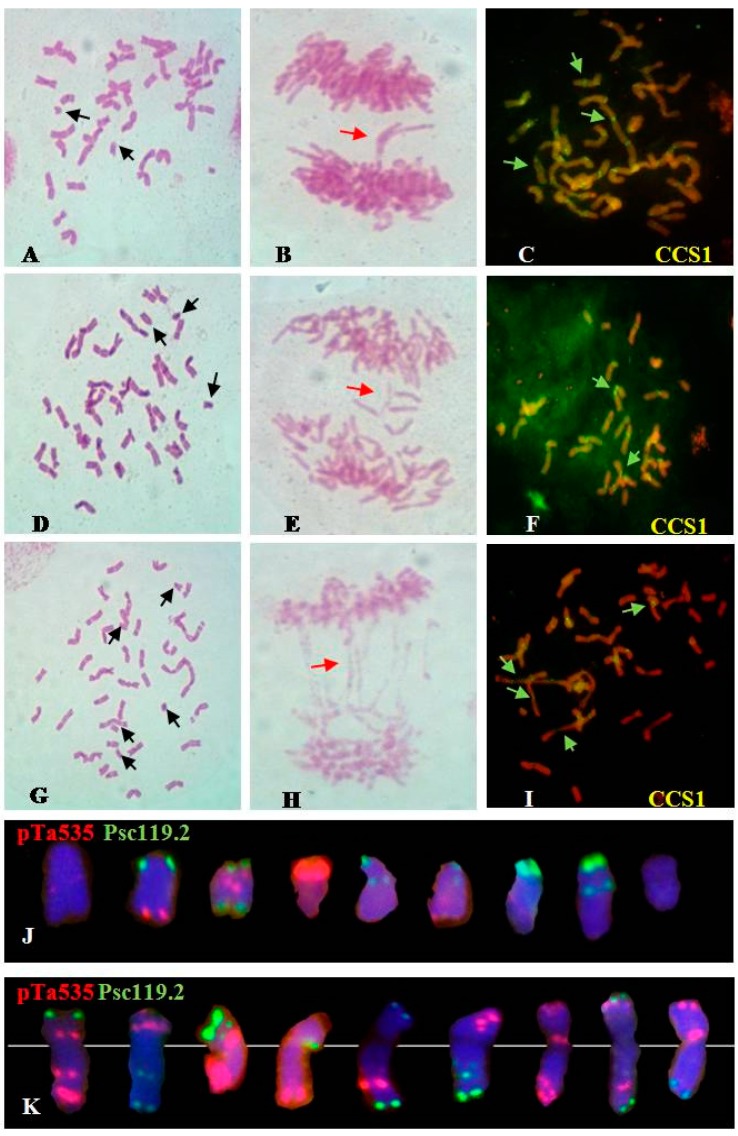
Summary of the irradiation-induced chromosomal aberrations in the M_0_ generation. (**A**–**C**): Feulgen staining and fluorescence *in situ* hybridization (FISH) analyses of M_0_ generation of 100 Gy group; (**D**–**F**): Feulgen staining and FISH analyses of M_0_ generation of 200 Gy group; (**G**–**I**): Feulgen staining and FISH analyses of M_0_ generation of 300 Gy group; (**J**,**K**): Fragments and translocations. Black arrows indicate fragments, red arrows indicate chromatin bridges, and green arrows indicate dicentrics. Oligo-CCS1 can be used as probe to investigate centromeric structure [15]. Chromosomes were counterstained with 4′-6-diamidino-2-phenylindole (blue).

M_0_ seeds from each dosage class (100, 200, and 300 Gy) were randomly selected and analyzed using Feulgen staining and ND-FISH. Abnormal mitotic behavior and large-scale structural aberrations of chromosomes such as deletions, translocations, dicentrics and chromatin bridges were observed in the three dosage groups (Figure 2). Normal chromosomes were recorded as N chromosomes (normal chromosomes), and significant aberrations, mainly involving chromosome fragments, were recorded as A chromosomes (abnormal chromosomes). The average chromosome number per cell was calculated; the corresponding chromosome numbers for the treated groups were as follows: 100 Gy group: 2*n* = 43.51 (43.24N + 0.27A); 200 Gy group: 2*n* = 42.26 (40.93N + 1.33A); and 300 Gy group: 2*n* = 40.21 (35.42N + 4.79A); Table 1. As expected, higher doses of γ-irradiation resulted in an increase in the number of chromosomal aberrations (A chromosomes).

**Table 1:** The frequency of normal chromosomes (N) and aberrations (A) in the M_0_ generation treated with γ-irradiation at doses of 100, 200, and 300 Gy.

γ-Irradiation (Gy)	No. of Cells Observed	No. of Chromosomes (per cell)	Percentage of Normal Chromosomes (%)	Percentage of Abnormal Chromosomes (%)
100	41	43.51	99.38	0.62
200	42	42.26	96.82	3.18
300	43	40.21	88.09	11.91

### 2.3. Chromosomal Aberrationsin M_1_ Plants

**Figure 3:**
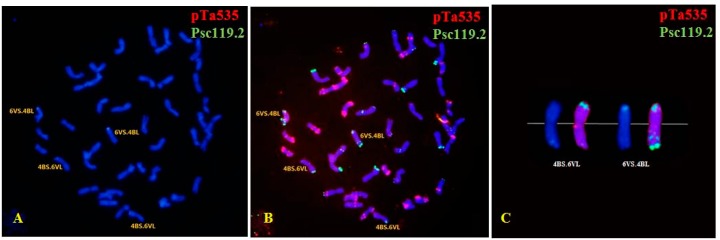

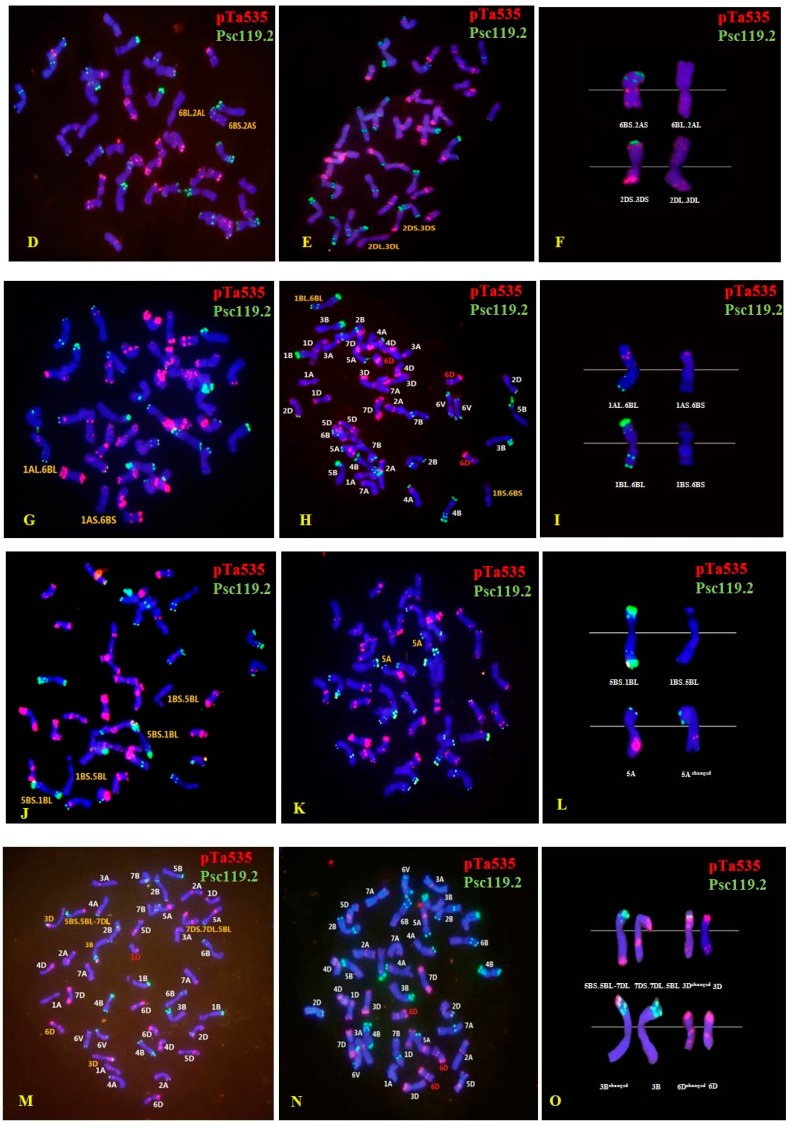
Fluorescence *in situ* hybridization (FISH) performed usingpTa535 (red) and pSc119.2 (green) as probes for chromosomes with structural changes and mutants in the M_1_ generation. (**A**,**B**) 4B.6V translocation; (**D**) 2A.6B translocation; (**E**) 2D.3D translocation; (**G**) 1A.6B translocation; (**H**) 1B.6B translocation, and 6D trisome; (**J**) 5B.1B translocation; (**K**) 5A aberrations; (**M**) 5B.7D translocation, 3D and 6D aberrations, and 1D monosome; (**N**) 6D trisome. Images (**C**,**F**,**I**,**L**,**O**) show enlargements of the FISH pattern of chromosomes involved in the structural changes. Chromosomes were counterstained with 4′-6-diamidino-2-phenylindole (blue).

In all, 101 M_1_ plants were successfully identified using ND-FISH; these included 48 M_1_ plants in the 100 Gy group; 47 in the 200 Gy group; and only six in the 300 Gy group (owing to infertility). Abnormal mitotic behavior and dicentric chromosomes were not detected in the M_1_ plants. In contrast, chromosomal loss and rearrangements were observed in all the three dosage groups. Both wheat chromosomes and *D. villosum* chromosome 6V were involved in variations of chromosomal structure and number. The mutation rate increased with an increase in the radiation dose: 19 plants (39.58%) of the 100 Gy group, 27 plants (57.45%) of the 200 Gy group, and 6 plants (100%) of the 300 Gy group. Of the 101 M_1_ plants, 39 contained structurally changed chromosomes; these included 29 wheat-wheat translocation plants and two wheat-*D. villosum* translocations (4B.6V). The wheat chromosomes showing structural variations were 1A, 2A, 4A, 5A, 1B, 2B, 3B, 4B, 5B, 6B, 7B, 1D, 2D, 3D, 4D, 6D and 7D (Figure 3 and Figure S1).

**Figure 4:**
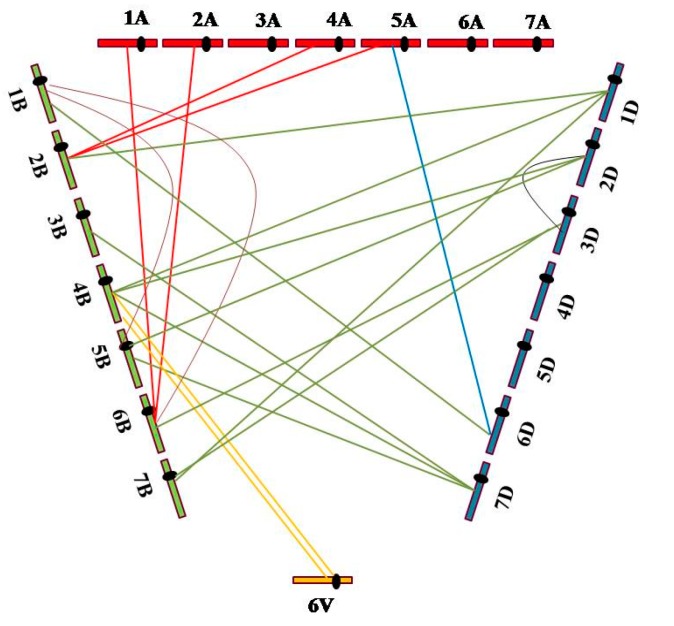
Distribution of interchromosomal translocation events on chromosomes of A, B, and D wheat genomes and the alien chromosome 6V from *Dasypyrum villosum*.

The FISH patterns of 31 plants carrying the translocation chromosomes revealed 21 interchromosomal translocation events that involved all three genomes (A, B, and D), as well as the 6V chromosome. Of the translocation events, four (19.05%) involved A and B genomes; one (4.76%), genomes A and D; 11 (52.38%), genomes B and D; two (9.52%), genome B and 6V; two (9.52%), different B genome chromosomes; and one (4.76%), different D genome chromosomes. Moreover, up to 42 breakages occurred on the chromosomes of the three wheat genomes and the 6V chromosome, including five (11.90%) breakages in the A genome, 21 (50%) in the B genome, 14 (33.33%) in the D genome and two (4.76%) on the 6V chromosome (Figure 4). These results indicated that the breakage frequency of the B genome was considerably higher than that of the A and D genomes, and that the B genome tended to recombine with the D genome.

Furthermore, other structural variations were identified. For instance, one line contained a changed 5A chromosome, whose long arm carried the weaker intercalary oligo-pTa535-1 signal (Figure 3K,L).Six lines were observed in the 300 Gy group: the 3B chromosome carrying an apparent terminal oligo-pTa535-1 signal on the short arm, a 6D chromosome with a weak terminal oligo-pTa535-1 signal on the short arm, and an extra fragment and a strong oligo-pSc119.2-1 signal on the end of 3DS (Figure 3M,O). One line carried two structurally changed 6B chromosomes without a terminaloligo-pSc119.2-1 signal on 6BL, but a strong terminal oligo-pSc119.2-1 signal on the end of the satellite (Figure S1G,I). In another plant, 4D chromosomes showed a significant terminal oligo-pSc119.2-1 signal on the long arm unlike that in the normal line (Figure S1H,I).

In addition, 19 plants showing variations in chromosome number, such as nullisomic lines, monosomic lines, and trisomic lines of 6D, were also found among the 101 M_1_ plants. These variants involved the three wheat genomes and chromosome 6V. In total, 26 events of chromosome loss were observed in the 19 plants, among which one event involved the A genome (3.85%); nine, the B genome (34.62%); 12, the D genome (46.15%); and four, chromosome 6V (15.38%). Chromosome 6D was associated with considerably higher loss frequencies (reaching 30.77%) than the other chromosomes, whereas the alien chromosome 6V produced the second highest loss frequencies (Figure 3M,N; Figure S1D).

## 3. Discussion

The effects of irradiationon the frequency of chromosomal aberrations, rate of germination, and changes in morphological and physiological traits have been previously documented [16,17,18,19]. In agreement with the findings of previous studies on wheat-*Aegilops biuncialis* amphiploids [19,20], those of the present study suggested the presence of translocations, fragments, and dicentrics in all three dosage groups (100, 200, and 300 Gy).Furthermore, the frequency of aberrances increased with an increase in the irradiation dose in the M_0_ generation of the irradiated line WD14. In M_1_ plants, the 200 Gy group showed a higher frequency of chromosomal variation (reaching 57.45%) than those in the 100 Gy group and higher plant survival rate than that of the 300 Gy group. Therefore, we found that 200 Gy was the most optimal dosage for treating dry seeds of bread wheat.

ND-FISH is a relatively new cytogenetic technique for the rapid detection of plant chromosomes without requiring prior denaturation of chromosomes [21] by using oligonucleotides as probes [15]. Our results confirmed that ND-FISH with oligonucleotides pSc119.2-1 and pTa535-1 as probes could be used to discriminate between wheat genomes and alien chromosome 6V from *D. villosum*. Thirty-nine plants with structural changes in their chromosomes were detected rapidly and accurately; of these, 31 plants showed interchromosomal translocations, of which 29 were wheat-wheat and two were wheat-*D. villosum*. Moreover, according to FISH patterns, the B genome showed more aberrations in M_1_, suggesting that the B genome chromosomes are more sensitive to irradiation. Chromosomal breakpoints are known to be mainly distributed in the heterochromatin [22,23] or the border area between them [24], resulting in a higher sensitivity of the heterochromatic regions to radiation-induced breakage [25]. According to C–banding analysis, the B genome is the most heterochromatic [26]. However, larger chromosomes show aberrations more frequently than smaller ones [27], and B genome has the largest DNA content for each of the three genomes of wheat [28]. Thus, both the highest heterochromatin content and large genome of the B genome were thought to be associated with the highest sensitivity to irradiation. Ma *et al*. [29] detected a significantly smaller number of putative interchromosomal translocation events in the D genome and suggested that, since the A and B genomes combined considerably earlier than the D genome, the interchromosomal rearrangements seem to have occurred at a similar frequency among the A, B, and D genomes. In this study, the frequency of interchromosomal events involving the D genome were significantly higher than those of the A genome after γ-irradiation. These results indicated the presence of a moderate discrepancy between spontaneous and induced interchromosomal translocations, and the D genome was confirmed to be more sensitive to irradiation than the A genome, likely due to the high number of DNA transposons in this genome [30]. Wheat-wheat translocation lines such as 5BS.7BS have been reported to possess high resistance to powdery mildew and durable resistance to yellow rust [31,32], implying that these lines could be useful genetic blocks for wheat breeding (with the exception of wheat-alien translocations such as 1RS.1BL and 6VS.6AL). In this study, we detected 29 wheat-wheat translocation plants; some of these as well as some plants of the M_1_ generation possessing favorable new traits (Figure S2) might be selected and would be useful for introduction in breeding programs.

In addition to structural aberrations, 19 plants showed variations in chromosome number. As expected, FISH confirmed the events of chromosome loss in the A, B, and D genomes. According to FISH analyses, the highest frequency of chromosome loss occurred on 6D rather than on the alien chromosome 6V. The highest frequency of 6D loss might have resulted from chromosomal functional redundancy.

Taken together, our results indicated the advantage of using ND-FISH with oligonucleotides as probes to detect irradiation-induced chromosomal aberrances. The interchromosomal translocations, chromosomal structural aberrances, and deletions detected in this study are expected to be stabilized, which would become a good genetic resource to carry useful traits in wheat breeding programs as well as to map genes on chromosomes.

## 4. Materials and Methods

### 4.1. Plant Materials

Common wheat, Chinese Spring (CS)-*D. villosum* nullisomic-tetrasomic (6A/6D) additional (6V) line (2*n* = 44), WD14, was selected from CS-*D. villosum* additional (6V) #3 line (2*n* = 44) and was kindly provided by Yang Zujun (University of Electronic Science and Technology of China, Chengdu, China). Dry seeds of WD14 were subjected to ^60^Coγ-irradiation at dosages of 100, 200, and 300 Gy (dose rate = 1.0 Gy/min) at the Institute of Biological and Nuclear Technology, Sichuan Academy of Agricultural Sciences, Chengdu, China. The seeds treated by ^60^Co-γ ray were germinated, and planted in field. The mutagenized generation (M_0_) was analyzed using Feulgen staining and ND-FISH, and the self-pollinated progenies (M_1_) were analyzed using ND-FISH to identify chromosomal aberrances involving the wheat genome and 6V chromosome of *D. villosum*.

### 4.2. Mitotic Studies

The preparation of root tips, and the protocols of mitotic studies were operated according to De Tomasi [33]. Mitosis behavior was analyzed using the acetocarmine squash method. Cytological observations were performed on an Olympus CX31 microscope (Olympus, Shanghai, China).

### 4.3. FISH Analysis

Chromosome preparations were made from root tips according to Kang *et al.* [34]. After were fixed in ethanol: glacial acetic acid (3:1, *v*/*v*), the root tips were squashed on microscope slides in 45% acetic acid. The slides were frozen in liquid nitrogen for one minute, and then the cover slips were removed quickly. The oligonucleotide probes pSc119.2-1, pTa-535-1, and CCS1were synthesized according to the method described by Tang *et al.* [15] (Invitrogen, Shanghai, China). *In situ* hybridization was performed according to the method described by Han *et al.* [35]. Microphotographs of FISH patterns were obtained using a Leica DM2500 microscope (Leica, Shanghai, China).

## Supplementary Materials

Supplementary materials can be found at http://www.mdpi.com/1422-0067/16/12/26134/s1.

## Abbreviations

CS: Chinese Spring; ND-FISH: non-denaturing fluorescence *in situ* hybridization.
