# Supplementary material for: Identification of Novel Chromosomal Aberrations Induced by 60Co-γ-Irradiation in Wheat-Dasypyrum villosum Lines
Source: Int J Mol Sci. 2015 Dec 14;16(12):29787–96. doi: 10.3390/ijms161226134 (PMC4691081; doi:10.3390/ijms161226134)
Supplement: Supplementary file 1 [file ijms-16-26134-s001.pdf]

Supplementary Materials: Identification of Novel Chromosomal Aberrations Induced by ^{60}Co-γ-irradiation in Wheat-*Dasypyrum villosum* Lines

Jie Zhang, Yun Jiang, Yuanlin Guo, Guangrong Li, Zujun Yang, Delin Xu and Pu Xuan

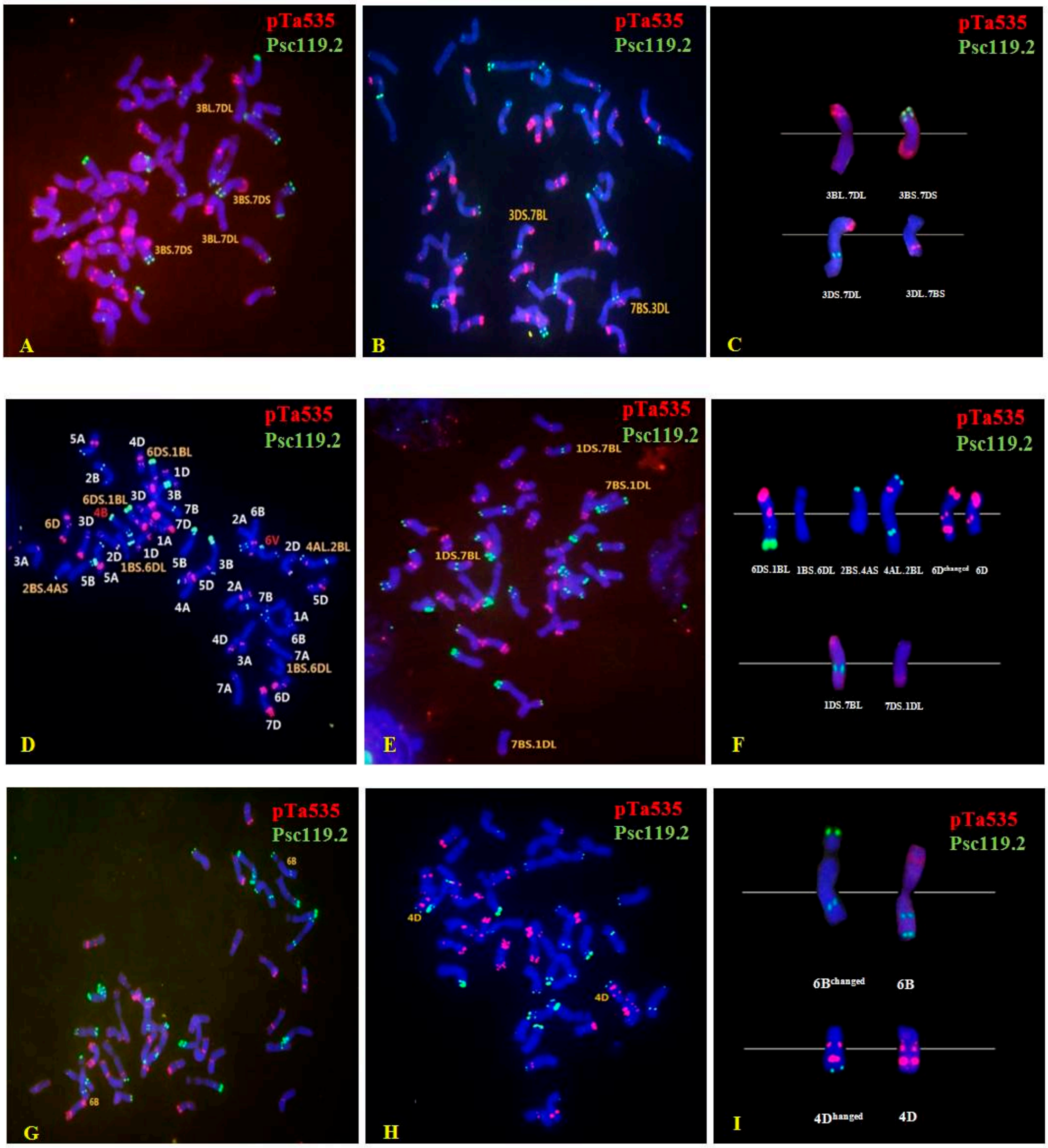

Figure S1. FISH, usingpTa535 (red) and pSc119.2 (green) as probes, for chromosomes with structural changes and number mutant plants from the M_1 generation. (**A**) 3B.7D translocation; (**B**) 3D.7D translocation; (**D**) 1B.6D and 4A.2B translocations,4B and 6V monosomes; (**E**) 1D.7B translocation; (**G**) 6B aberrance; (**H**) 4D aberrance; (**C**,**F**,**I**,) Enlargement of the FISH pattern of the chromosomes involved in the structural change. Chromosomes were counterstained with 4'-6-diamidino-2-phenylindole (blue).

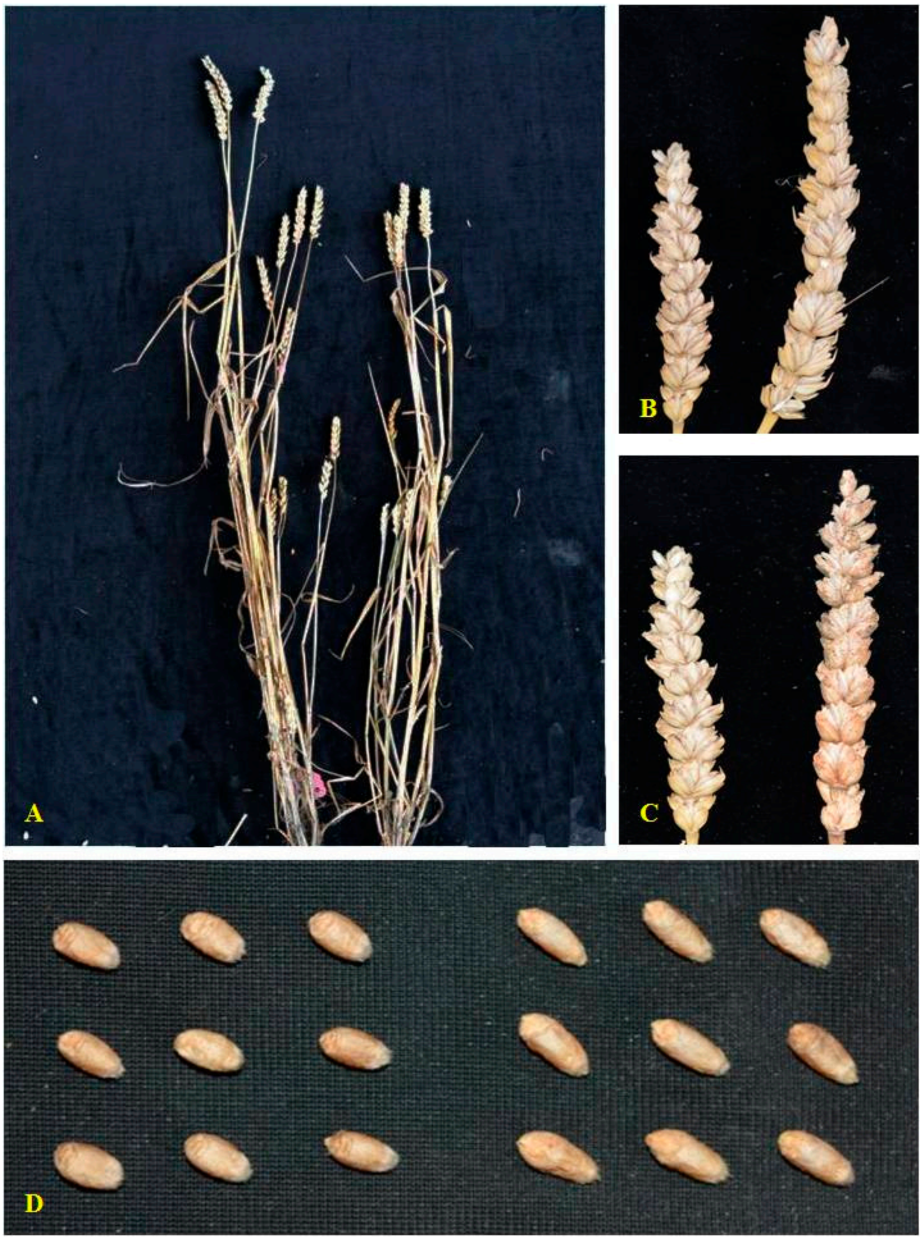

Figure S2. Themorphology of plants in M_1 generation. (**A**) Dwarf plant (4B.1D translocation); (**B**,**C**) Long spikes (c is 6B.3D translocation); (**D**) Long seeds. The plant (**A**), spike (**B**,**C**), seeds (**D**) on the left are of control check (CK, untreated WD14), and those on the right are from a treated plant possessing favorable traits.
